# Clinical Characteristics, Etiology, and Prognostic Scores in Patients with Acute Decompensated Liver Cirrhosis

**DOI:** 10.3390/jcm12175756

**Published:** 2023-09-04

**Authors:** Hoor Al Kaabi, Abdullah M. Al Alawi, Zubaida Al Falahi, Zakariya Al-Naamani, Said A. Al Busafi

**Affiliations:** 1 Internal Medicine Residency Training Program, Oman Medical Specialty Board, Muscat 130, Oman; hooralkaabi11@gmail.com (H.A.K.); zubaida.alfalahi@gmail.com (Z.A.F.); busafis@squ.edu.om (S.A.A.B.); 2 Department of Medicine, Sultan Qaboos University Hospital, Muscat 123, Oman; zakriya2006@hotmail.com; 3 College of Medicine and Health Sciences, Sultan Qaboos University, Muscat 123, Oman

**Keywords:** chronic liver disease, cirrhosis, liver transplantation, organ failure, viral hepatitis, alcohol

## Abstract

Background: Chronic liver disease and cirrhosis contribute significantly to global mortality, with limited improvements despite medical advancements. This study aims to evaluate acute decompensation of liver cirrhosis characteristics, etiology, and survival outcomes in Oman. In addition, we examined the accuracy of prognostic scores in predicting mortality at 28 and 90 days. Methods: We conducted a retrospective analysis of 173 adult patients with acute decompensation of liver cirrhosis at Sultan Qaboos University Hospital in Oman. We collected demographic, clinical, and biochemical data, including etiology, prognostic scores (CTP, MELD-Na, CLIF-C), and health outcomes. Results: Alcohol (29.5%), hepatitis C (27.75%), and hepatitis B (26.74%) were the predominant causes of liver cirrhosis in our cohort. Hepatic encephalopathy, mechanical ventilation, and admission to the intensive care unit were strongly associated with an increased mortality rate. The 1-year readmission rate stood at 42.2%. Liver transplantation was performed in 4.1% of cases. The overall mortality rate was approximately 40% during the follow-up period, and the cumulative 28-days and 90-days mortality rates were 20.8% and 25.4%, respectively. Prognostic scores (CTP, MELD-Na, CLIF-C) effectively predicted 28- and 90-day mortality, with CLIF-C demonstrating superior performance (AUROC 0.8694 ± 0.0302 for 28-day mortality and AUROC 0.8382 ± 0.0359 for 90-day mortality). Conclusion: Alcohol and viral hepatitis are the leading causes of liver cirrhosis in our study. Hepatic encephalopathy is a significant predictor of poor outcomes. Prognostic scores (CTP, MELD-Na, CLIF-C) have valuable predictive abilities for short-term mortality. These findings highlight the importance of public strategies to reduce alcohol consumption and the need for the comprehensive management of liver cirrhosis in Oman. Early diagnosis and intervention can improve clinical outcomes and support the establishment of a national organ transplantation program to address the healthcare challenge effectively.

## 1. Introduction

Chronic liver disease and cirrhosis rank is one of the leading cause of death globally, and unfortunately, liver disease-related mortality has shown no improvement over the past 30 years, despite advancements in medical care, hepatology, and post-liver transplant outcomes [1,2]. The impact of chronic liver disease is not only seen in the significant morbidity and mortality, but also in the considerable healthcare costs it incurs worldwide [3]. Alcohol, viral hepatitis and non-alcoholic fatty liver disease (NAFLD) are the most common causes [4]. Cirrhosis progresses silently until increased portal pressure and declining liver function lead to clinical symptoms. The European Association for the Study of the Liver (EASL) defines acute decompensation of liver cirrhosis as the occurrence of overt clinical manifestations such as ascites, encephalopathy, or bleeding, or the sudden onset of cirrhosis-related complications like renal failure, coagulopathy, or bacterial infection [5]. Hepatocellular carcinoma is a devastating complication of liver cirrhosis [6]. Liver transplantation remains the main curative therapy for liver cirrhosis [4]. Other treatment modalities are directed towards the etiologies, symptoms and complications of liver cirrhosis [7]. The determination of prognostic predictors remains very crucial in the stratification of cirrhotic patients. This helps in estimating overall survival, directing the optimal therapy, and eventually identifying the candidates for liver transplantation. Thus, numerous prognostic scores have been proposed. Child–Turcotte–Pugh (CTP) is widely applied in predicting the 1-year survival rate in patients with cirrhosis [7]. The Mayo End-Stage Liver Disease (MELD) score has been validated in determining the severity of liver dysfunction, 3-month mortality, and the suitability for liver transplantation [4]. The Chronic Liver Failure Consortium—Acute-on-Chronic Liver Failure (CLIF-C ACLF) score has been introduced recently and found to be superior to CTP and MELD scores in predicting short-term (28-day) mortality as well as medium-term (90-day) mortality in both ICU patients and those who were admitted in the ward [8,9].

Studies from the Middle East and North Africa Region (MENA) on chronic liver disease are sparse [10,11,12,13,14]. The objectives of this study include evaluating demographic and clinical characteristics, determining the etiology of liver cirrhosis, assessing short-term and long-term survival outcomes, and examining the accuracy of prognostic scores in predicting mortality at 28 and 90 days. Additionally, the study aims to identify factors associated with increased mortality and investigate the occurrence of hepatocellular carcinoma as a complication of liver cirrhosis in this population from single center from the MENA region.

## 2. Materials and Methods

### 2.1. Study Design and Population

This study is a retrospective study conducted at Sultan Qaboos University Hospital in Oman, a multi-specialty, advanced care, teaching and research center [15]. The study included adult patients admitted to the hospital between January 2015 and December 2021, who had acute decompensation of liver cirrhosis. For patients with multiple admissions, their initial admission with acute decompensation of liver cirrhosis was considered as the index admission. Acute decompensation of liver cirrhosis was defined as hospitalization due to hepatic encephalopathy, upper gastrointestinal bleeding, ascites, hepatorenal syndrome, worsening liver function, kidney function, and coagulation profile.

### 2.2. Data Collection

Patients’ electronic health records were utilized to gather relevant demographic, clinical, and biochemical data. The data collected from the index admission included demographic characteristics, admission diagnosis, pertinent comorbidities (such as heart failure and diabetes mellitus), the cause of liver cirrhosis, length of hospital stay, need for intensive care admission, relevant hematological and biochemical test results (e.g., international normalized ratio (INR), sodium levels), and the treatment administered. Additionally, the study involved the calculation of the Child–Turcotte–Pugh (CTP) score, with Grade A representing 5–6 points, Grade B representing 7–9 points, and Grade C indicating ≥10 points. The Model for End-Stage Liver Disease (MELD) score and the Chronic Liver Failure-Consortium (CLIF-C) score were also determined. Furthermore, outcomes variables, such as 28- and 90-day readmission rates, were extracted from electronic health records. If needed, these outcomes were further verified through phone follow-up.

### 2.3. Statistical Analysis

Categorical variables were expressed as numerical values and percentages, while continuous variables were presented as means for normally distributed data or as medians with interquartile range (IQR) for non-normally distributed data. To compare continuous variables between the two groups, the student t-test was employed for normally distributed variables, and the Wilcoxon rank sum test was utilized for non-normally distributed variables. The chi-squared test was applied to assess the relationship between categorical variables. All relevant variables were fitted into stepwise backward regression analysis to identify independent predictors of mortality. The predictive accuracy of CTP, MELD, and CLIF-C scores for survival was evaluated by calculating the area under the receiver operating characteristics (AUROC) curve. Mortality was analyzed as a time-to-event outcome using the Kaplan–Meier method, and hazard ratios with corresponding 95% confidence intervals (CIs) were computed. Statistical significance was established at a two-sided *p*-value below 0.05. Statistical analyses were conducted using Stata v. 17.0 software package (StataCorp LLC, College Station, TX, USA).

## 3. Results

We identified a total of 173 patients admitted with acute decompensation of liver cirrhosis at SQUH between 1 January 2015 and 31 December 2021. Baseline demographic, clinical and biochemical characteristics are shown in Table 1.

The mean age was 58 ± 13.8 years; 71.7% of patients (*n* = 124) were males. The most common comorbidities were hypertension (44.5%, *n* = 77), diabetes mellitus (43.4%, *n* = 75), and heart failure (20.8%, *n* = 36). The most frequent causes of liver cirrhosis were alcohol (29.5%, *n* = 51), followed by hepatitis C virus (27.8%, *n* = 48), and hepatitis B virus (26.7%, *n* = 46). The length of hospital stay was 7 (IQR:4–12) days.

There were 34 (19.7%) patients admitted to the intensive care unit (ICU), and 31 (17.9%) patients required mechanical ventilation. Ascites (64.2%, *n* = 111) and varices (49.1%, *n* = 85) were the most common reason for admission. Regarding treatment, lactulose (77.46%, *n* = 134), diuretics (65.90%, *n* = 114), and beta blockers (48.6%) were commonly prescribed for patients. In terms of prognostic scores, the CTP score was 9 (IQR: 7–11), the MELD-Na score was 18 (13–25), and the CLIF-C score was 41 (IQR 35–48), as calculated during the index admission.

**Figure 1:**
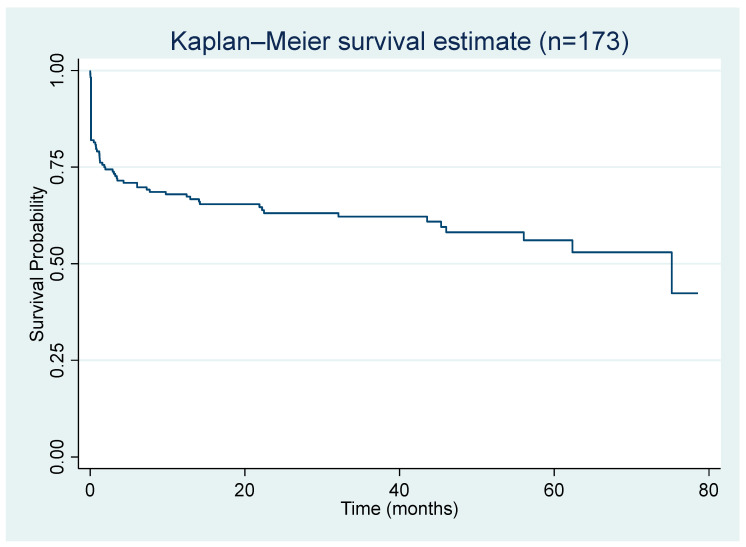
Kaplan–Meier survival analysis demonstrates survival after the index admission with acute decompensated liver cirrhosis (*n* = 173).

The median follow-up period was 20.8 (2.9–44.9) months. The 1 year readmission rate was 42.20% (*n* = 73). Also, 22.5% of patients (*n* = 39) developed hepatocellular carcinoma, and only seven patients (4.1%) had liver transplantation. The median survival time was 75.2 months (Figure 1).

The overall mortality rate was approximately 40% (*n* = 69; 95% CI: 32.8–47.4%) during the follow-up period, and the cumulative 28- and 90-day mortality rates were 20.8% (*n* = 36; 95% CI:15.4–27.6%) and 25.4% (*n* = 44, 95% CI: 19.5–32.5%), respectively.

Longer length of hospital stay (10.5 (IQR:6.5–23.5) vs. 6 (IQR: 4–11), *p* < 0.01), hepatic encephalopathy (75% vs. 29.9%, *p* < 0.01 ), ICU admission (66.7% vs. 7.3%, *p* < 0.01), need for mechanical ventilation (61.1% vs. 6.6%, *p* < 0.01), use of lactulose (94.4% vs. 73.0%, *p* < 0.01), and high CTP score (10 (IQR: 9–12) vs. 8 (IQR: 7–10), *p* < 0.01), MELD-Na score (24 (IQR: 18–29) vs. 17 (12–24)) and CLIF-C score (52 (45–59) vs. 39 (33–44), *p* < 0.01) were associated with increased 28-day mortality. On the other hand, the use of Beta blockers was associated with reduced 28-day mortality (33.3% vs. 52.6%, *p =* 0.04) (Table 2).

Longer length of hospital stay (10.5 (IQR:5.5–23.5) vs. 6 (IQR: 4–11), *p* < 0.01), hepatic encephalopathy (68.2% vs. 29.5%, *p* < 0.01 ), ICU admission ( 61.4% vs. 5.4%, *p* < 0.01), need for mechanical ventilation (54.6% vs. 5.4%, *p* < 0.01), use of lactulose (90.9% vs. 72.87%, *p* < 0.01), and high CTP score (10(IQR:9- 12) vs. 8 (IQR:7–10), *p* < 0.01), MELD-Na score (24 (IQR:18- 27.5) vs. 16.5 (12–23)) and CLIF-C score (50 (44–58) vs. 38 (33–43), *p* < 0.01) were associated with increased 90 days mortality (Table 3).

**Figure 2:**
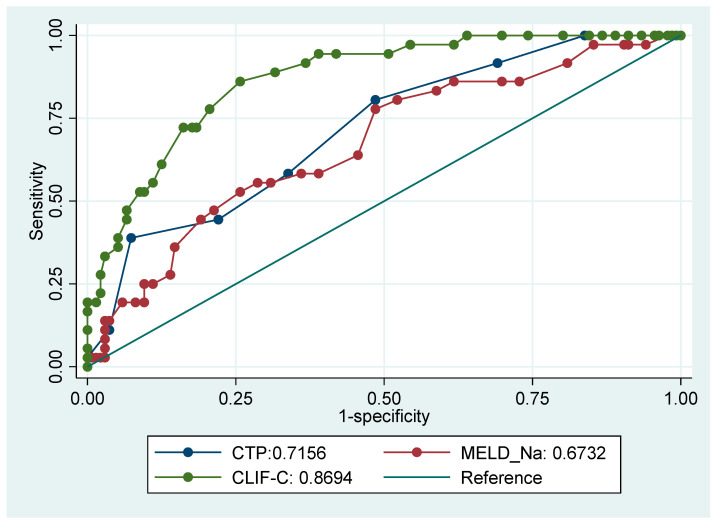
Receiver operating characteristic (ROC) curve for mortality comparing prognostic scores at 28 days.

**Figure 3:**
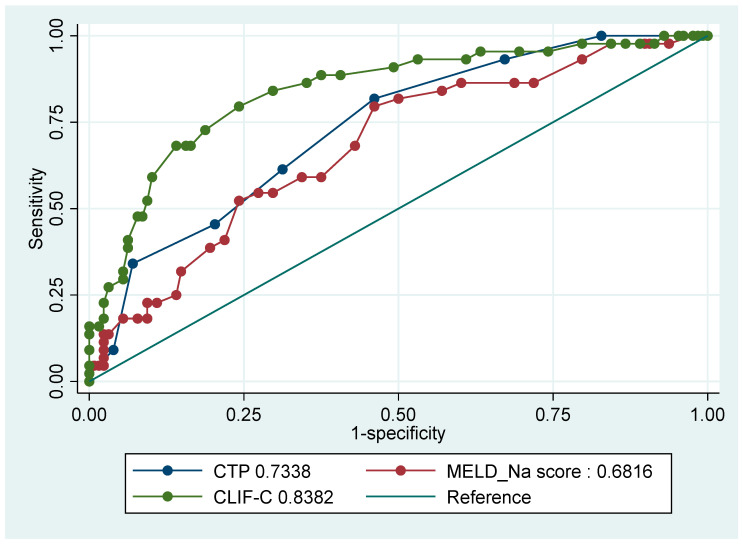
Receiver operating characteristic (ROC) curve for mortality comparing prognostic scores at 90 days.

Figure 2 and Figure 3 illustrate the receiver operating characteristic (ROC) curves for the mortality of several prognostic scores (CTP, CLIF-C, MELD-Na score) at 28 and 90 days from the index admission.

All scores were able to predict mortality for patients admitted with acute decompensation significantly better than the reference line at 28 and 90 days. CLIF-C was significantly superior to CTP, MELD and MELD-Na in predicting 28-day (AUROC0.8694 ± 0.0302, 95% CI 0.81021–0.92856) and 90-day mortality (AUROC 0.8382 ± 0.0359, 95% CI 0.76778–0.90854) (Table 4 and Table 5).

## 4. Discussion

This study is one of very few studies from the Middle East and North Africa (MENA) reporting patients’ characteristics and etiology of liver cirrhosis [10,11,12,13,14]. In addition, we have reported short- and long-term survival outcomes following admission with decompensated liver cirrhosis. The study reported the accuracy of three prognostic scores (CTP, CLIF-C, MELD-Na score) in predicating the 28- and 90-day mortality.

This retrospective study revealed a predominance of male patients, accounting for approximately two-thirds of the cases, which aligns with findings reported in certain regions of the world [16,17]. This observation may be attributed to the higher prevalence of alcohol disorders among men as compared to women in Oman [18,19].

The mean age of the study population was 58 ± 13.8 years, and the average length of hospital stay was 7 (IQR: 4–12) days. These results are consistent with previously reported findings from a similar healthcare setting, where the mean hospital stay was 7.97 ± 4.28 years [16].

Identifying the cause of liver cirrhosis is crucial for effective treatment, as many causes are preventable and treatable. In our study, alcohol consumption emerged as the most frequent attributable risk factor for liver cirrhosis, accounting for 29.48% of all cases. The prevalence of alcohol consumption among patients with liver cirrhosis has varied in previously reported literature. For instance, a retrospective cohort study conducted in the Department of Medical Gastroenterology, Thiruvananthapuram, found alcohol to be the identified cause in 91% of cases presenting with the first episode of decompensation between 2008 and 2012 [20]. Studies from Vienna and France reported alcohol-related liver disease as the most common etiology of liver cirrhosis, accounting 56.6% and 66.6% of the cases, respectively [21,22]. However, it is crucial to emphasize that regarding alcohol, it predominantly emerges as the primary cause for the admission of patients who present with acute decompensation, rather than a leading cause of cirrhosis itself in our study. Also, alcohol is a recognized precipitant of acute decompensation in alcohol-related cirrhosis, as well as in cases associated with other cirrhosis-related causes, including patients with viral hepatitis, as reported in previous literature [23]. Our study also reported other common causes of liver cirrhosis, with hepatitis C virus and hepatitis B virus accounting for 27.75% and 26.74%, respectively. Non-alcoholic fatty liver disease (NAFLD) was found in 13.95% of cases, and in 5.78% of cases, the cause of liver cirrhosis remained unclear even after thorough investigations. In a study conducted at two Hepatology Centers in Bogota among patients with decompensated liver cirrhosis from 2010 to 2014, the main etiologies in this series were NAFLD (25.5%), alcoholic cirrhosis (14.8%), hepatitis C infection (14.6%), and autoimmune cirrhosis (10%) [24].

Liver cirrhosis exhibits a diverse clinical spectrum. In our study, the decompensation event during the index admission was commonly manifested as ascites in 64.16% of patients, followed by varices in 49.13% of cases, and hepatic encephalopathy in 39.31%. Similar findings were demonstrated in a retrospective study conducted at the Vienna General Hospital between 2010 and 2017, which evaluated the clinical characteristics and outcomes of 173 patients after their first decompensation event of liver cirrhosis, revealing that 72.3% had ascites, followed by 20.2% with varices during their first decompensation event [20]. These results are also supported by another prospective study conducted in India [16].

The treatment of liver cirrhosis is centered on treating the etiology, recognizing the complications, and identifying the precipitating factors. Numerous studies clearly demonstrated the efficacy of lactulose and its importance in treating and preventing further episodes of hepatic encephalopathy [25]. Strangely, in our study, the use of lactulose has been observed to be associated with worse clinical outcome and overall mortality. In total, 40 out of 44 patients who died after 90 days (91% of overall 90-days mortality) were commenced on lactulose. This finding might be explained by the fact that starting patients on lactulose goes parallel with the development of hepatic encephalopathy, which has its own impact on the overall clinical outcome and mortality. Another explanation is that lactulose discontinuation or non-adherence has been found to trigger breakthrough hepatic encephalopathy, which again has an impact on clinical outcome and survival. On the contrary, it was also reported that lactulose overuse can induce dehydration, which is a well-known trigger for liver decompensation, particularly hepatic encephalopathy [26].

Diuretics are the mainstay pharmacological treatment for ascites in cirrhotic patients. In our study, diuretics use has no statistical significance in relation to mortality rate. This result might be affected by many factors, including medication non-adherence, which probably underestimates the role of diuretics in the disease course and overall outcome. Certain studies also reported that patients might respond differently to diuretics and some of them are prone to develop side effects of diuretics—mainly dehydration and acute kidney injury [27]. This might result in using a suboptimal dose to minimize the side effect and hence limit the role of diuretics in preventing fluid accumulation and improving the overall outcome. In addition to this, a larger sample size or longer duration might be required to detect the effects of diuresis in this population.

Non-selective beta blockers are widely used as primary and secondary prophylaxis for variceal bleeding in cirrhotic patients, as they act on reducing portal blood flow [28]. In our retrospective analysis, 84 (48.55%) patients were commenced on beta blockers and the survival rate among them was 52.55%. The impact of beta blockers on overall survival was also assessed in a meta-analysis of 12 selected randomized trials. The latter showed that the mean survival rate at 2 years was 74% in patients treated with beta blockers, with a 5.4% mean improvement rate [29]. The difference in survival rate between both studies might be attributed to the duration of both studies.

Cirrhosis of different etiologies can eventually predispose one to hepatocellular carcinoma. In our study, 39 (22.54%) patients developed HCC. Liver transplantation remains the only curative option for liver cirrhosis. In our analysis, only seven (4.05%) patients underwent liver transplant. This might be related to organ shortages and the absence of donors, as many patients are placed on the waitlist. This problem seems to be international, and was also reported in Germany, where more than 50% of candidates were placed on the waitlist in 2011 [30].

The prognosis of liver cirrhosis is primarily influenced by its cause and the presence of complications. Patients with decompensated liver cirrhosis are particularly prone to complications and often require hospitalization. The high rate of readmission among cirrhotic patients is widely acknowledged, placing a burden on patients, their families, the healthcare system, and the economy. In our study, the 1-year readmission rate for our population was 42%. Prior estimates of readmission rates in cirrhotic patients have ranged from 13% to 37% within 30 days of hospital discharge [31]. A study conducted at the University of Michigan, US, showed that 69% of cirrhotic patients experience at least one readmission, with a median time to first readmission of 67 days [32]. Another population-based study in Canada reported readmission rates of 24.6% within <90 days and 75.4% within >90 days. The variation in readmission rates among different studies could be attributed to differences in the sites of readmission, as many patients seek medical attention from various healthcare institutions [33]. Furthermore, the heterogeneity of patients with liver cirrhosis in terms of disease etiology, severity, comorbidities, and medication compliance may also influence the risk of readmission.

The overall mortality rate was approximately 40% within 90 days. In this study, hepatic encephalopathy was strongly associated with 28- and 90-days mortality, accounting for 75% of death within 28 days from the first event of decompensation. These data are in agreement with those from a previously reported retrospective study conducted in the United Status, which concluded that the development of hepatic encephalopathy in cirrhotic patients is a sign associated with short life expectancy [34].

Liver cirrhosis can present with multiorgan failure requiring admission to an intensive care unit (ICU). According to our retrospective analysis, the ICU admission rate was (19.65%, 34 patients). Amongst them, 24 patients died, representing 66.67% of overall 28-days mortality, whereas only 7 (5.43%) patients survived after 90 days. This result was comparable to what was reported in one population-based cohort study in Taiwan, where associations between liver cirrhosis and increased 30-day mortality were significant in both sexes and every age group [35]. In another prospective dual-center non-transplant ICU study in London, evaluating the prevalence of ICU mortality in 137 cirrhotic patients, ICU mortality rate was 38% over a period of 20 months. This high rate of mortality is unsurprising and has several explanations. The requirement of ICU admission marks severe advanced disease that has its own consequences and is significantly associated with multiorgan failure. This includes hepatorenal syndrome, which is a marker of advanced liver cirrhosis and associated with only 15% survival rate [36]. Overwhelming sepsis is another burden that poses a higher risk for morbidity and increased mortality by 4-fold in cirrhotic patients, according to previous studies [37]. Notably, our analysis showed that out of the 173 patients, 31 required invasive mechanical ventilation, and of them, 22 patients died within 28 days, while only 7 patients remained alive after 90 days. This mortality rate was higher compared to the figure reported in London, UK, where the application of mechanical ventilation did not show any association with mortality [38]. This might be highly affected by the indication of intubation, as respiratory failure is known to be associated with worse clinical outcome compared to GI bleeding, as demonstrated in this study [38].

As liver cirrhosis remained a global health challenge and places a high burden on the patient, health system, and health financing, many scores have been proposed to predict the disease severity and mortality in patients with liver cirrhosis. Acute-on-chronic liver failure (ACLF) refers to the rapid deterioration of liver function coupled with the failure of other organs, leading to high short-term mortality in individuals with pre-existing chronic liver disease (CLD). However, there is no universally agreed-upon set of diagnostic criteria for ACLF, and its distinction from ordinary decompensation of CLD has often been a subject of debate [39]. CLIF-C was validated primarily to stratify the risk of mortality in ACLF patients, and was found to be superior to the MELDs and MELD-Na in predicting mortality [40]. In the present study, we have included all patients with acute decompensation of liver cirrhosis defined as hospitalization resulting from hepatic encephalopathy, upper gastrointestinal bleeding, ascites, hepatorenal syndrome, or deterioration in liver function, kidney function, or coagulation profile in patients who are known to have liver cirrhosis.

In this study, the mean CTP, MELD-Na and CLIF scores of the patient were reported as 9, 18 and 41, respectively. Almost similar findings were reported in a retrospective in Venna, in which patients’ scores were 9.3, 16.9 and 53.7, respectively [20]. All scores were able to predict mortality at 28 and 90 days better than the reference range. Furthermore, the CLIF-C score is superior to MELD and CTP scores in predicating 28- and 90-days mortality.

This study provides insights into liver cirrhosis in a country from the MENA region, highlighting patient characteristics, etiology, and survival outcomes. Alcohol emerged as a prominent risk factor, and hepatitis C and hepatitis B viruses were common causes. Clinical manifestations during decompensation included ascites, varices, and hepatic encephalopathy. Lactulose use showed unexpected associations with worse outcomes. Diuretics’ impact on mortality was inconclusive. Non-selective beta blockers demonstrated potential in preventing variceal bleeding. Liver transplantation remained limited due to organ shortage. High readmission rates posed challenges, and ICU admissions were linked to increased mortality rates. Prognostic scores (CTP, CLIF-C, MELD-Na) proved effective in predicting mortality.

We acknowledge some potential limitations of the present study. First, as it is a retrospective study and depends mainly on the documentation obtained from the patient records, some information could not be collected due to a lack of documentation. This could potentially affect the overall results and clinical outcome. Second, a larger sample size and longer duration might be required to achieve better outcomes. Lastly, the study was carried out in a single center, which might limit the generalizability of the findings.

## 5. Conclusions

The study’s results align with international findings, highlighting alcohol and viral hepatitis as the leading admission causes for patients presenting with acute decompensation of liver cirrhosis in Oman. Public strategies to reduce alcohol consumption are crucial. Hepatic encephalopathy emerges as a significant predictor of poor outcomes. The superiority of the CLIF-C score in predicting short-term mortality emphasizes its integration into daily practice. These findings highlight the burden of liver cirrhosis on the nation, urging the implementation of future strategies to enhance clinical outcomes. Moreover, the study supports the establishment of a national organ transplantation program to address this healthcare challenge effectively.

## Figures and Tables

**Table 1:** Baseline characteristics, clinical findings, and biochemical profiles of patients (*n* = 173).

Characteristic (*n* = 173)	*n* (%) Unless Specified Otherwise
Age (years)	58 ± 13.8
Male (*n*)	124 (71.7%)
Weight (kg)	69.3 (84.0–60.0)
BMI	27.7 (31.8–22.4)
Comorbidity	
Hypertension	77 (44.5%)
Diabetes Mellitus	75 (43.4%)
Heart failure	36 (20.8%)
Chronic kidney disease	20 (11.6%)
Smoking	30 (17.3%)
Aetiology of liver cirrhosis	
Alcohol	51 (29.5%)
Hepatitis B virus (HBV)	46 (26.7%)
Hepatitis C virus (HCV)	48 (27.8%)
Non-Alcoholic Fatty Liver Disease (NAFLD)	24 (14.0%)
Autoimmune hepatitis	5 (2.9%)
Wilson disease	1 (0.6%)
Hemochromatosis	0 (%)
Others	18 (10.4%)
Cryptogenic	10 (5.8%)
Index admission	
Length of hospital stay (days)	7.0 (4–12)
Spontaneous bacterial peritonitis (SPB)	15 (8.7%)
Hepatic encephalopathy	68 (39.3%)
Ascites	111 (64.2%)
Varices related admission	85 (49.1%)
Intensive care unit (ICU) admission	34 (19.7%)
Mechanical ventilation	31 (17.9%)
Hematological and biochemical profile	
Hemoglobin (g/dL)	10.2 ± 2.5
Platelets × 10^9^/L	154.5 (211.5–99.5)
White cell count × 10^9^/L	7.2 (10.7–5.1)
International normalized ratio (INR)	1.32 (1.5–1.16)
Serum creatinine level (μmol/L)	72 (106–57)
Serum sodium (μmol/L)	135 (131–138)
Serum potassium (μmol/L)	4.3 (3.9–4.8)
Alanine aminotransferase (ALT) U/L	37 (24–70)
Albumin (g/L)	30 (25–34)
Alkaline phosphatase (ALP) U/L	136 (100–220)
Aspartate aminotransferase (AST) U/L	63 (42–134)
Bilirubin (μmol/L)	34 (17–79)
Gamma-glutamyl transferase (GGT) U/L	230 (75–481)
Child–Turcotte–Pugh (CTP) score	9 (7–11)
Model for End-Stage Liver Disease (MELD)-Na score	18 (13–25)
Chronic Liver Failure Consortium (CLIF)-C	41(35–48)
Treatment	
Beta blockers	84 (48.6%)
Diuretics	114 (65.9%)
Lactulose	134 (77.5%)
Rifaximin	4 (2.3%)
Liver transplant	7 (4.1%)
Health outcome	
Follow up duration (months)	20.8 (2.9–44.9)
Readmission within 1 year	73 (42.20%)
Number of admission within 1 year	1 (0–1)
Hepatocellular carcinoma	39 (22.54%)
Mortality	69 (39.88%)

**Table 2:** Relevant demographic characteristics, clinical profile and 28-day mortality.

	Total Number of Patients (*n* = 173)	28-Days Mortality (*n* = 36)	No 28-Days Mortality (*n* = 137)	*p* Value
Age	58 ±13.8	62.0 ± 13.7	57.0 ± 13.7	0.0496
Male (*n*)	124 (71.7%)	30 (83.33%)	94 (68.61%)	0.081
Length of hospital stay (days)	7 (4–12)	10.5 (6.5–23.5)	6 (4–11)	0.0030
Weight (kg)	69.3 (84–60)	70 (60–81.65)	69.2 (60.8–85)	0.5144
BMI	27.7 (31.8–22.4)	28.1 (22.1–30.5)	27.5 (22.6–32)	0.4386
Hypertension	77 (44.51%)	12 (33.33%)	65 (47.45%)	0.129
Diabetes Mellitus	75 (43.35%)	14 (38.89%)	61 (44.53%)	0.544
Cardiac disease	36 (20.81%)	9 (25.00%)	27 (19.71%)	0.486
Chronic kidney disease (CKD)	20 (11.56%)	5 (13.89%)	15 (10.95%)	0.623
Smoking	30 (17.34%)	10 (27.78%)	20 (14.60%)	0.063
Alcohol	51 (29.48%)	13 (36.11%)	38 (27.74%)	0.327
Hepatitis B virus	46 (26.74%)	7 (19.44%)	39 (28.68%)	0.266
Hepatitis C virus	48 (27.75%)	13 (36.11%)	35 (25.55%)	0.208
Non-Alcoholic Fatty Liver Disease (NAFLD)	24 (13.95%)	5 (14.29%)	19 (13.87%)	1.000
Spontaneous bacterial peritonitis (SPB)	15 (8.67%)	4 (11.11%)	11 (8.03%)	0.519
Hepatic encephalopathy	68 (39.31%)	27 (75.00%)	41 (29.93%)	0.000
Ascites	111 (64.16%)	26 (72.22%)	85 (62.04%)	0.257
Varices	85 (49.13%)	12 (33.33%)	73 (53.28%)	0.033
Intensive care unit (ICU) admission	34 (19.65%)	24 (66.67%)	10 (7.30%)	0.000
Mechanical ventilation	31 (17.92%)	22 (61.11%)	9 (6.57%)	0.000
Beta blockers	84 (48.55%)	12 (33.33%)	72 (52.55%)	0.040
Diuretics	114 (65.90%)	24 (66.67%)	90 (65.69%)	0.913
Lactulose	134 (77.46%)	34 (94.44%)	100 (72.99%)	0.006
Rifaximin	4 (2.31%)	1 (2.78%)	3 (2.19%)	1.000
Child–Turcotte–Pugh (CTP) score	9 (7–11)	10 (9–12)	8 (7–10)	0.0001
Model For End-Stage Liver Disease (MELD)-Na score	18 (13–25)	24 (18–29)	17 (12–24)	0.0014
Chronic Liver Failure Consortium-C (CLIF-C)	41 (35–48)	52 (45–59)	39 (33–44)	0.0000

Backward stepwise regression analysis showed that hepatic encephalopathy was associated with increased risk of 28-days mortality (odd ratio: 7.8; *p* < 0.01; 95% CI (3.0–20.3)), while the use of Beta blockers was associated with reduced risk of 28-days mortality ((OD): 0.38; *p =* 0.04; 95% CI (0.15–0.95)).

**Table 3:** Relevant demographic characteristics, clinical profile and 90-days mortality.

	Total Number of Patients (*n* = 173)	90-Days Mortality (*n* = 44)	No 90-Days Mortality (*n* = 129)	*p* Value
Age	58 ±13.8	61.4 ± 15.6	56.9 ± 13	0.0604
Male (*n*)	124 (71.7%)	34 (77.27%)	90 (69.77%)	0.439
Length of hospital stay (days)	7 (4–12)	10.5 (5.5–23.5)	6 (4–11)	0.0046
Weight (kg)	69.3 (84–60)	65 (59.1–80.3)	70 (61–86.8)	0.1490
BMI	27.7 (31.8–22.4)	28.1 (22.1–30.4)	27.5 (22.4–32.4)	0.4850
Hypertension	77 (44.51%)	16 (36.36%)	61 (47.29%)	0.208
Diabetes Mellitus	75 (43.35%)	17 (38.64%)	58 (44.96%)	0.465
Cardiac diseases	36 (20.81%)	11 (25.00%)	25 (19.38%)	0.428
CKD	20 (11.56%)	5 (11.36%)	15 (11.63%)	1.000
Smoking	30 (17.34%)	11 (25.00%)	19 (14.73%)	0.120
Alcohol	51 (29.48%)	14 (31.82%)	37 (28.68%)	0.694
Hepatitis B virus	46 (26.74%)	8 (18.18%)	38 (29.69%)	0.137
Hepatitis C virus	48 (27.75%)	16 (36.36%)	32 (24.81%)	0.139
Non-Alcoholic Fatty Liver Disease (NAFLD)	24 (13.95%)	6 (13.95%)	18 (13.95%)	1.000
Spontaneous bacterial peritonitis (SPB)	15 (8.67%)	5 (11.36%)	10 (7.75%)	0.462
Hepatic encephalopathy	68 (39.31%)	30 (68.18%)	38 (29.46%)	0.000
Ascites	111 (64.16%)	32 (72.73%)	79 (61.24%)	0.170
Varices	85 (49.13%)	16 (36.36%)	69 (53.49%)	0.050
Intensive care unit (ICU) admission	34 (19.65%)	27 (61.36%)	7 (5.43%)	0.000
Mechanical ventilation	31 (17.92%)	24 (54.55%)	7 (5.43%)	0.000
Hepatocellular carcinoma (HCC)	39 (22.54%)	13 (29.55%)	26 (20.16%)	0.198
Beta blocker	84 (48.55%)	17 (38.64%)	67 (51.94%)	0.127
Diuretics	114 (65.90%)	29 (65.91%)	85 (65.89%)	0.998
Lactulose	134 (77.46%)	40 (90.91%)	94 (72.87%)	0.012
Child–Turcotte–Pugh (CTP) score	9 (7–11)	10 (9–12)	8 (7–10)	0.0000
Model For End-Stage Liver Disease (MELD)-Na score	18 (13–25)	24 (18–27.5)	16.5 (12–23)	0.0003
Chronic Liver Failure Consortium-C (CLIF-C)	41 (35–48)	50 (44–58)	38 (33–43)	0.0000

Backward stepwise regression analysis showed that hepatic encephalopathy (OR: 4.6; *p* < 0.01; 95% CI (2.0–10.4)) and length of hospital stay during the index admission (OR: 1.04; *p =* 0.02; 95% CI: 1.01–1.1) were associated with increased risk of 90-days mortality.

**Table 4:** Receiver operating characteristic (ROC) curve for mortality comparing prognostic scores at 28 days.

Prognostic Score (*n* = 172)	ROC	Standard Error	95% CI
Child–Turcotte–Pugh (CTP) score	0.7156	0.0461	0.62524–0.80593
Model For End-Stage Liver Disease (MELD)-Na score	0.6732	0.0506	0.57402–0.77238
Chronic Liver Failure Consortium-C (CLIF-C)	0.8694	0.0302	0.81021–0.92856

**Table 5:** Receiver operating characteristic (ROC) curve for mortality comparing prognostic scores at 90 days.

Prognostic Score (*n* = 172)	ROC	Standard Error	95% CI
Child–Turcotte–Pugh (CTP) score	0.7338	0.0409	0.65370–0.81399
Model For End-Stage Liver Disease (MELD)-Na score	0.6816	0.0457	0.59215–0.77113
Chronic Liver Failure Consortium-C (CLIF-C)	0.8382	0.0359	0.76778–0.90854

## Data Availability

Data are available from the corresponding author on reasonable request.
